# Supplementary material for: Endosomal NOX2 oxidase exacerbates virus pathogenicity and is a target for antiviral therapy
Source: Nat Commun. 2017 Jul 12;8:69. doi: 10.1038/s41467-017-00057-x (PMC5507984; doi:10.1038/s41467-017-00057-x)
Supplement: Supplementary file 1 — Supplementary Information [file 41467_2017_57_MOESM1_ESM.pdf]

File name: Supplementary Information
Description: Supplementary Figures and Supplementary Tables

File name: Peer Review File
Description:

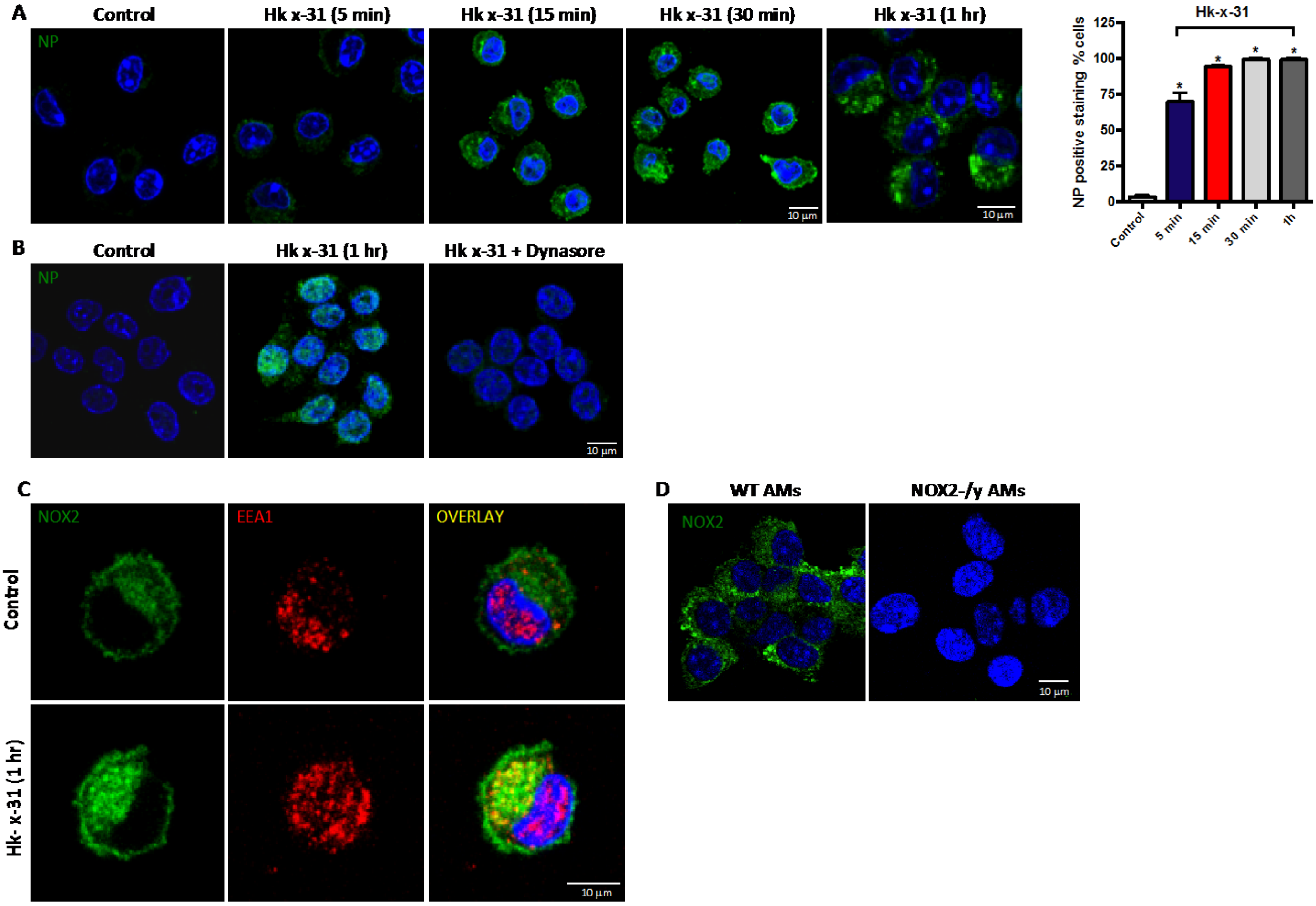

Supplementary Figure 1. Dynamin-dependent endocytosis of influenza A virus and entry into early endosomes expressing NOX2 oxidase.

(A, B) Confocal microscopy of WT mouse primary alveolar macrophages that were infected with the HKx31 strain of influenza A virus (MOI of 10 at various time points) in the absence and presence of the dynamin inhibitor, Dynasore (100 µM), and then labeled as indicated with antibody to influenza A virus nucleoprotein (NP) and 4',6'- diamidino-2-phenylindole (DAPI) (n=5). **(C)** Alveolar macrophages infected with influenza A virus as in **A)**, and then labeled with the early endosome antigen 1 (EEA1) antibody and NOX2 antibody. **(D)** WT and NOX2$^{-/y}$ alveolar macrophages were labeled with the NOX2 antibody and DAPI. **(A-D)** Images are representative of > 100 cells analysed over each experiment. Original magnification X100. Data are shown as mean ± SE. One-way ANOVA followed by Dunnett's *post hoc* test for multiple comparisons. * P<0.05 compared to control.

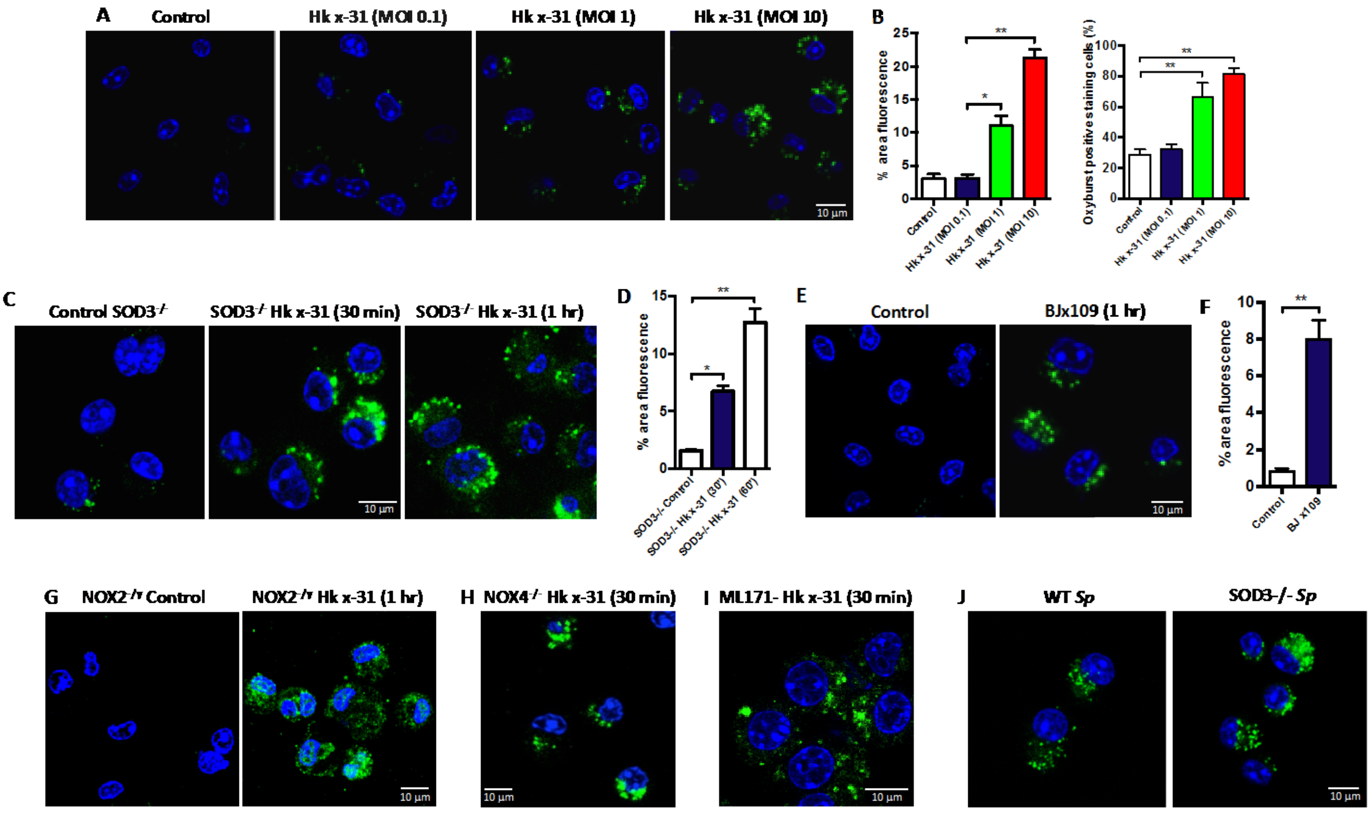

Supplementary Figure 2. Influenza A viruses induce dose-dependent elevations in endosomal NOX2 oxidase-dependent ROS production.

(A, B) Endosomal ROS production in wild-type (WT) mouse primary alveolar macrophages as assessed by OxyBURST (100 µM) confocal fluorescence microscopy in the absence or presence of various doses of HKx31 virus (MOI of 0.1, 1 and 10) and labeled with DAPI (n=5). **(C, D)** Endosomal ROS production following infection with the HKx31 virus in alveolar macrophages taken from superoxide dismutase 3 deficient ($SOD3^{-/-}$) mice (n=5). **(E, F)** Endosomal ROS production in WT alveolar macrophages following infection with the BJx109 virus (n=6). **(G)** Influenza nucleoprotein fluorescence in alveolar macrophages taken from $NOX2^{-/y}$ mice demonstrating that influenza internalization is unaffected by the expression of NOX2 (n=3). **(H and I)** Endosomal ROS production in **H)** $NOX4^{-/-}$ mouse primary alveolar macrophages or **I)** WT mouse primary alveolar macrophages treated with the NOX1 inhibitor ML171 (100nM) as assessed by OxyBURST fluorescence microscopy in the presence of HKx31 virus and labeled with DAPI (n=5). **(J)** Phagosomal ROS production to *Streptococcus pneumoniae (Sp)* as assessed by OxyBURST fluorescence microscopy in WT or $SOD3^{-/-}$ alveolar macrophages (n=3). **(A, C, E, G, H, I, J)** Images are representative of >100 cells analysed over each experiment. Original magnification X100. All data are represented as mean ± SEM. **(B and D)** One-way ANOVA followed by Dunnett's *post hoc* test for multiple comparisons. * P<0.05 comparisons indicated by horizontal bars. **P<0.01. **(F)** Unpaired t-test; statistical significance taken when the P<0.05. ** P<0.01

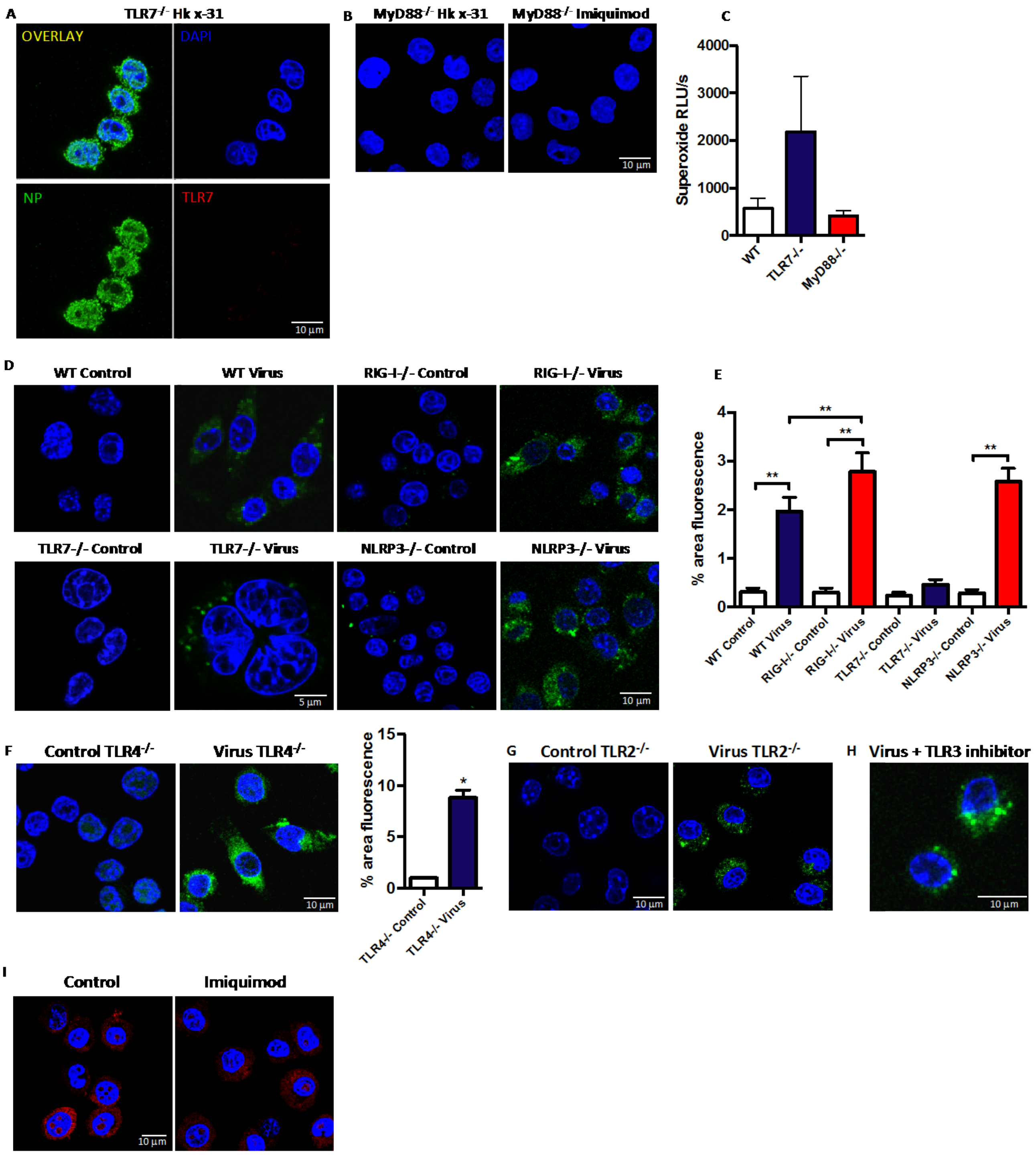

Supplementary Figure 3. Endosomal ROS production to influenza A virus is dependent on MyD88 but independent of RIG-I, NLRP3, TLR2, TLR3, and TLR4.

(A) TLR7$^{-/-}$ immortalized bone marrow-derived macrophages (BMDMs) were infected with HKx31 influenza A virus ((MOI of 10) and then stained with anti-TLR7 antibody and anti nucleoprotein (NP) antibody, and then DAPI (n=3). **(B)** MyD88$^{-/-}$ immortalized BMDMs were infected with HKx31 influenza A virus ((MOI of 10) or exposed to imiquimod (10 µg/mL) and endosomal ROS production was assessed by OxyBURST (100 µM) fluorescence microscopy (n=3). **(C)** Phorbol-dibutyrate-stimulated, NOX2 oxidase-dependent superoxide production in WT, TLR7$^{-/-}$ and MyD88$^{-/-}$ immortalized bone marrow-derived macrophages measured by L-O12 (100 µM)-enhanced chemiluminescence (n=3); RLU/s= relative light units/sec. **(D-G)** Endosomal ROS production in WT, RIG-I$^{-/-}$, TLR7$^{-/-}$, and NLRP3$^{-/-}$ and **F)** TLR4$^{-/-}$ or **G)** TLR2$^{-/-}$ BMDMs, as assessed by OxyBURST fluorescence microscopy in the absence or presence of HKx31 virus and co-labeled with DAPI (n=3-5). **(H)** WT mouse primary alveolar macrophages were infected with HKx31 virus (MOI of 10) in the presence of the TLR3 inhibitor (50 µM, n=3). **(I)** WT mouse primary alveolar macrophages were treated with imiquimod (10µg/mL) for 60 mins and mitochondrial superoxide measured by MitoSOX confocal fluorescence microscopy and co-labeled with DAPI (n=3). **(A, B, D, F, G, H and I)** Images are representative of >100 cells analyzed over each experiment. Original magnification X100. All data are represented as mean ± SEM. **(E)** One-way ANOVA followed by Dunnett's *post hoc* test for multiple comparisons. * P<0.05 and **P<0.01 comparisons indicated by horizontal bars. **(F)** Unpaired t-test; statistical significance taken when the P<0.05.

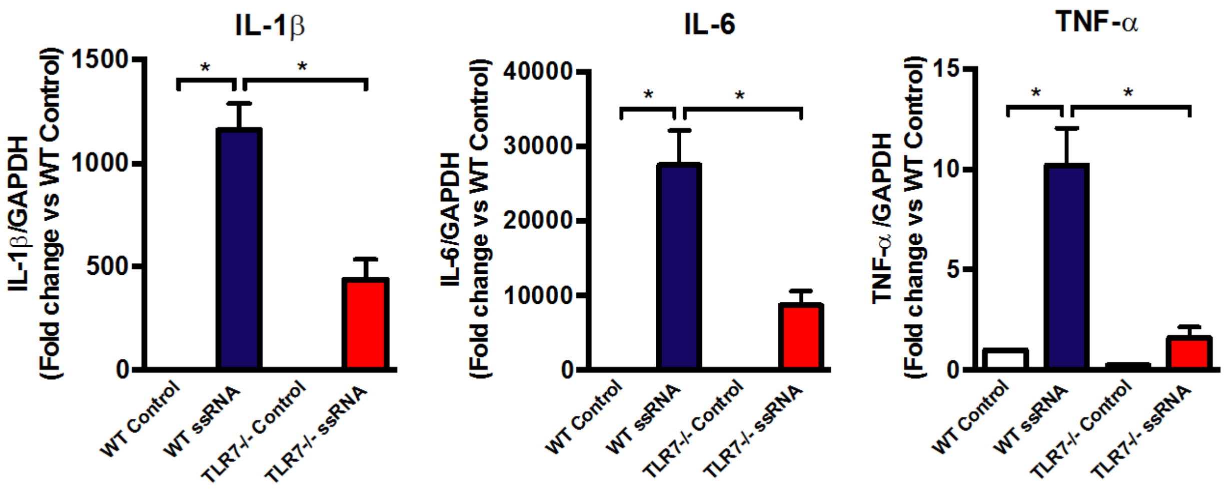

Supplementary Figure 4. Single stranded RNA (ssRNA) drives IL-1β, TNF-α and IL-6 mRNA expression *via* a TLR7-dependent mechanism in macrophages.
WT and TLR7$^{-/-}$ immortalized bone marrow derived macrophages were treated with ssRNA lyoVec (100 μM) and cytokine expression was determined by QPCR after 24 h. Kruskal-Wallis test with Dunn's *post hoc* for multiple comparisons. All data are represented as mean ± SEM. Statistical significance was taken when the P<0.05 (n=6).

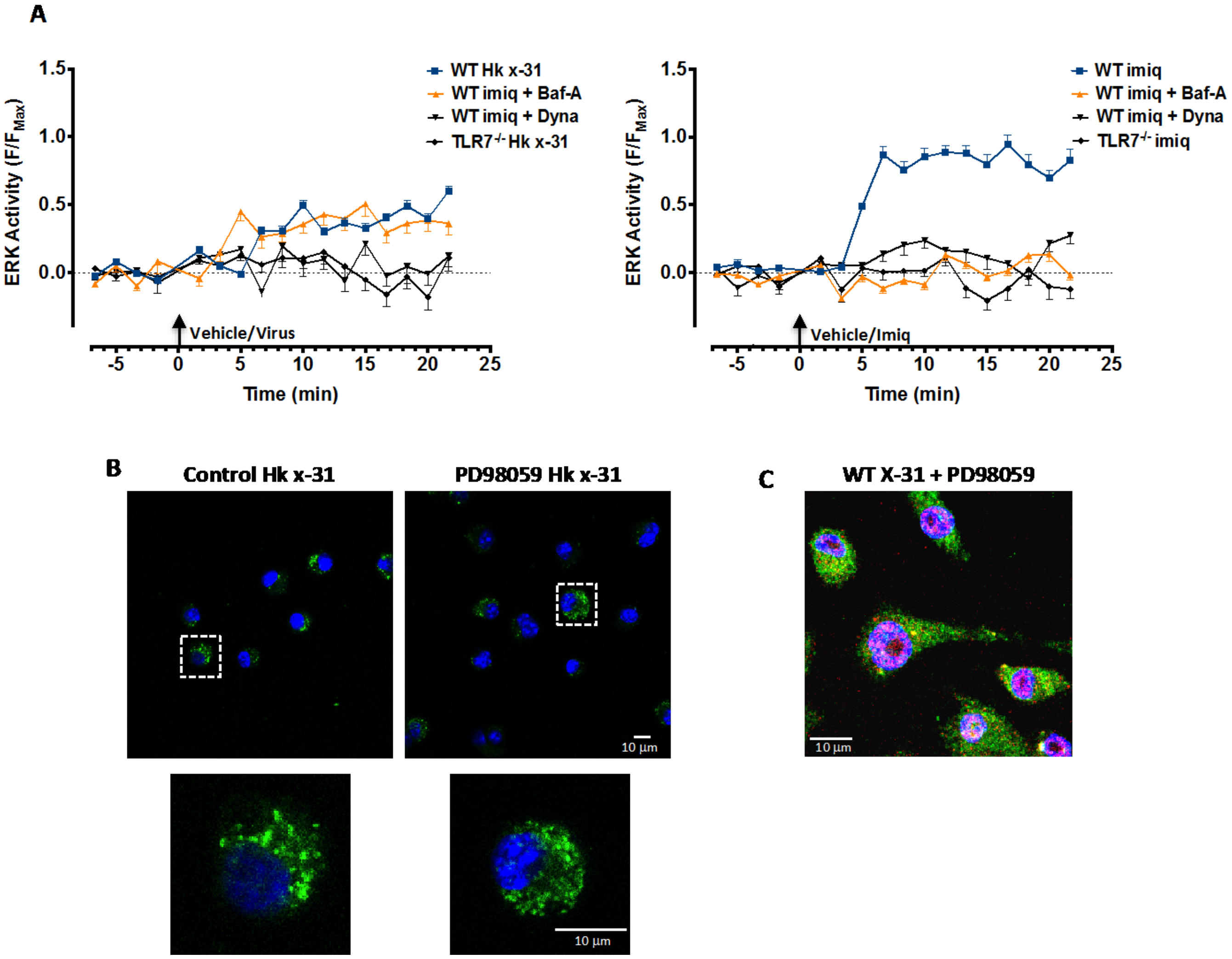

Supplementary Figure 5. Influenza A virus and imiquimod trigger ERK1/2-phosphorylation in a TLR7-dependent manner whereas ERK does not contribute to activation of NOX2 oxidase.

(A) Cytosolic ERK activity as assessed by high content ratiometric FRET imaging analysis in WT and TLR7$^{-/-}$ immortalized bone marrow-derived macrophages (BMDMs). Cells were either treated with vehicle controls or with bafilomycin A (Baf-A; 100 nM) or Dynasore (Dyna; 100 µM) and then exposed for 25 min to HKx31 influenza A virus (MOI of 10) or imiquimod (imiq; 10 µg/ml).All data are represented as mean ± SEM (n=3). **(B)** Endosomal superoxide production to HKx31 influenza A virus in WT alveolar macrophages as assessed by OxyBURST (100 µM) fluorescence microscopy in the absence or presence of the MEK inhibitor PD98059 (30 µM). **(C)** Immunofluorescence microscopy for assessment of NOX2 and p47phox association. WT immortalized BMDMs were infected with HKx31 virus, (MOI of 10) in the presence of PD98059 (30 µM) and then labeled with antibodies to NOX2 and p47phox. All images are representative of >100 cells analyzed over each experiment. Original magnification X100. (n=4).

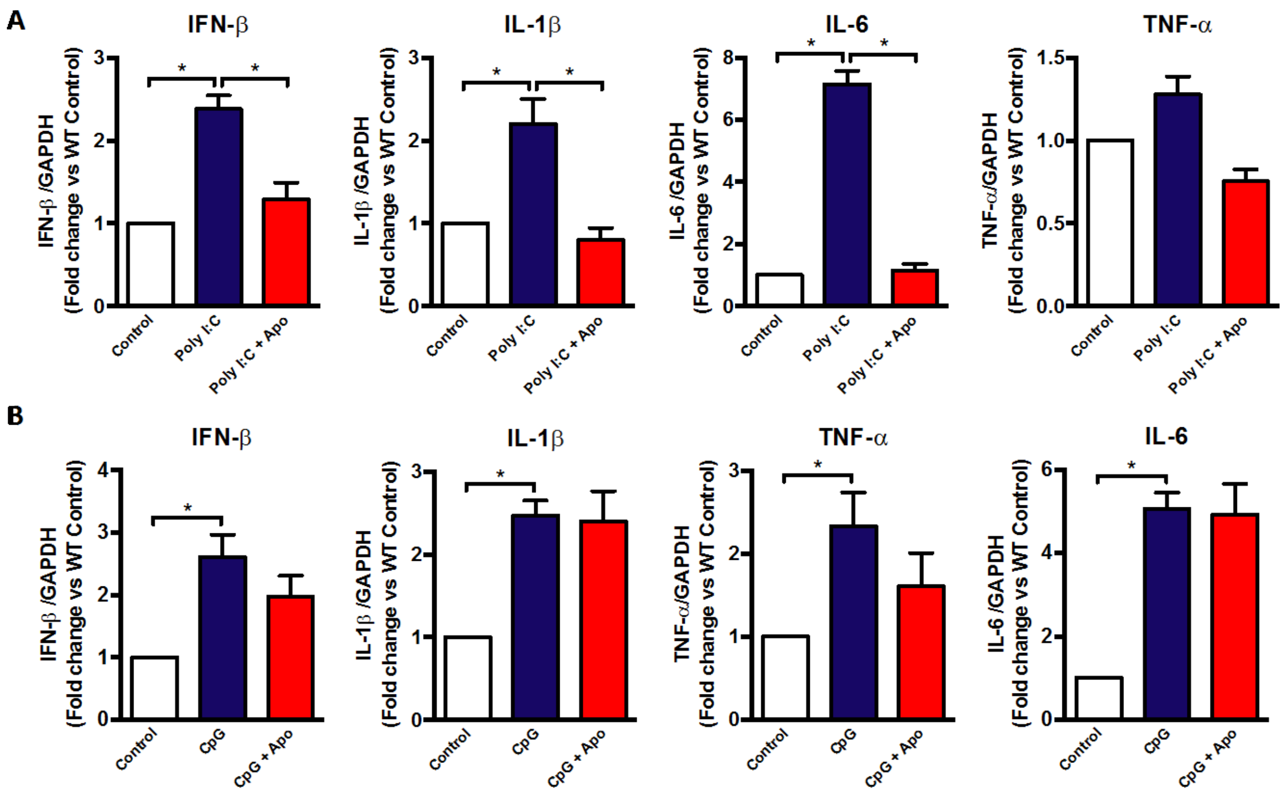

Supplementary Figure 6. Effect of NOX2 oxidase inhibition on cytokine expression by TLR3 agonist, poly I:C. or TLR9 agonist CpG. WT immortalized bone marrow derived macrophages were treated with **A)** poly I:C (25 μg/mL) or **B)** CpG (10μg/mL) in the absence or presence of apocynin (Apo; 300 μM) and cytokine expression was determined by QPCR after 24 h (n=6). Responses are relative to GAPDH and then expressed as a fold-change above controls. All data are represented as mean ± SEM. Kruskal-Wallis test with Dunn's *post hoc* for multiple comparisons. Statistical significance was taken when the P<0.05. * P<0.05.

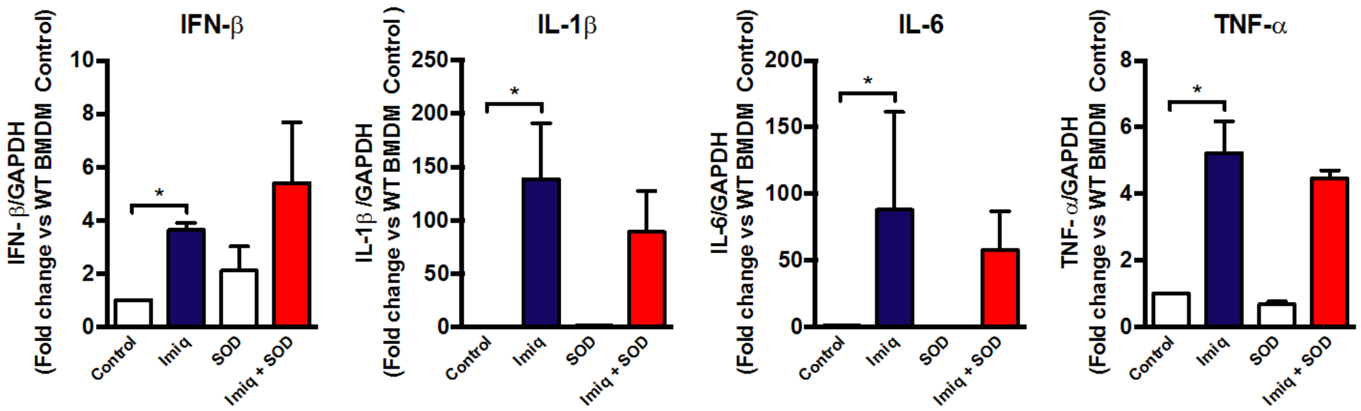

Supplementary Figure 7. TLR7-dependent cytokine production to imiquimod is unaffected by endosomal superoxide production.

WT immortalized bone marrow derived macrophages were treated with imiquimod (Imiq; 10 μg/mL) in the absence or presence of superoxide dismutase (SOD; 300 U/mL) and cytokine expression was determined by QPCR after 24 h. Responses are relative to GAPDH and then expressed as a fold-change above WT controls. All data are represented as mean ± SEM. Kruskal-Wallis test with Dunn's *post hoc* for multiple comparisons. Statistical significance was taken when the $P<0.05$. * $P<0.05$ (n=6).

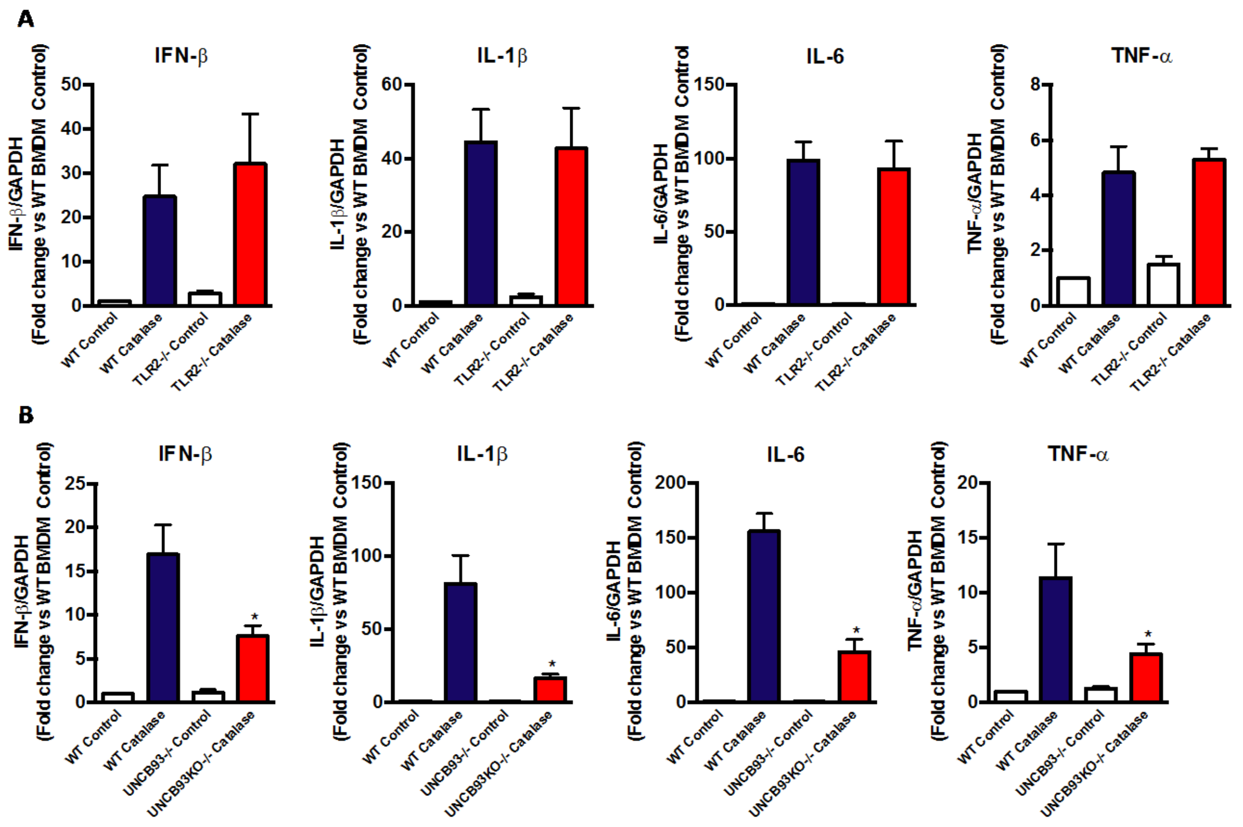

Supplementary Figure 8. Catalase-dependent elevation in cytokine expression is suppressed by deletion of UNCB93 but not by TLR2 deletion.

(A) WT and TLR2$^{-/-}$ immortalized bone marrow derived macrophages (BMDMs) were treated with catalase (1000 U/mL) for 1hr and cytokine mRNA expression determined by QPCR after 24 h (n=6). **(B)** WT and UNCB93$^{-/-}$ immortalized BMDMs were treated with catalase (1000 U/mL) for 1hr and cytokine mRNA expression determined by QPCR after 24 h (n=6). Responses are relative to GAPDH and then expressed as a fold-change above WT controls. All data are represented as mean ± SEM. Kruskal-Wallis test with Dunn's *post hoc* for multiple comparisons. Statistical significance was taken when the P<0.05. * P<0.05.

```
            C36             C51
TLR3        ------------------------MRQTLPCIY---------FWGGLLPF-------GML    20
TLR9        -------MGFCRSA----LHP---LS-----LLVQAIMLAMTLALGTLPA-------FLP    34
TLR7        -----------------MVFPMWTLKRLILILF-NIILISKLLGARWFPK-------TLP    35
TLR8        MKESSLQNSSCSLGKETKKENMFLQSSMLTCIFLLISGSCELCAEENFSR-------SYP    53
TLR5        ----------------------------MGDHLDLL--LGVVLMAGPVFG-------IPS    23
TLR4        ---------------------MMSASRLAGTL---I--PAMAFLSCVRPE------SWEP    28
TLR2        ----------------------------MP----HT--LWMVWVLGVIISLSKEESSNQA    26
TLR10       -------------------------MRLIRNIYIFC--SIVMTAEGDAPE----------    23
TLR1        ----------------------------MTSIFHFA--IIFMLILQIRIQ----------    20
TLR6        ---------------------MTKDKEPIVKSFHFV--CLMIIIVGTRIQ----------    27

            C36             C51
TLR3        CASSTTKCTVSHEVADCSHLKLTQVPD----DLPTNITVLNLTHNQLRRLPAANFTRYSQ    76
TLR9        CEL-QP-----HGLVNCNWLFLKSVPHFSMAAPRGNVTSLSLSSNRIHHLHDSDFAHLPS    88
TLR7        CDV-TLDVPKNHVIVDCTDKHLTEIPG----GIPTNTTNLTLTINHIPDISPASFHRLDH    90
TLR8        CDE-KK--QNDSVIAECSNRRLQEVPQ----TVGKYVTELDLSDNFITHITNESFQGLQN    106
TLR5        CSF-----DGRIAFYR--FCNLTQVPQ----VL-NTTERLLLSFNYIRTVTASSFPFLEQ    71
TLR4        CVE-----VVPNITYQCMELNFYKIPD----NLPFSTKNLDLSFNPLRHLGSYSFFSFPE    79
TLR2        SLS-----CDRNGICKGSSGSLNSIPS----GLTEAVKSLDLSNNRITYISNSDLQRCVN    77
TLR10       -LP-----EERELMTNCSNMSLRKVPA----DLTPATTTLDLSYNLLFQLQSSDFHSVSK    73
TLR1        -LS-----EESEFLVDRSKNGLIHVPK----DLSQKTTILNISQNYISELWTSDILSLSK    70
TLR6        -FS-----DGNEFAVDKSKRGLIHVPK----DLPLKTKVLDMSQNYIAELQVSDMSFLSE    77
                                     :  :*             * :: * :  :   .:

                   C98 C100    C112
TLR3        LTSLDVGFNTISK--------------LEPELCQKLPMLKVLNLQHNELSQLSDKTFAFC    122
TLR9        LRHLNLKWNCPPVGLSPMH--FPCHMTIEPSTFLAVPTLEELNLSYNNIMTVP----ALP    142
TLR7        LVEIDFRCNCVPIPLGSKNNMCIKRLQIKPRSFSGLTYLKSLYLDGNQLLEIPQ---GLP    147
TLR8        LTKINLNHNPNVQHQNGNPGIQSNGLNITDGAFLNLKNLRELLLEDNQLPQIPS---GLP    163
TLR5        LQLLELGSQYTPL-------------TIDKEAFRNLPNLRILDLGSSKIYFLHPDAFQGL    118
TLR4        LQVLDLSRC-EIQ-------------TIEDGAYQSLSHLSTLILTGNPIQSLALGAFSGL    125
TLR2        LQALVLTSN-GIN-------------TIEEDSFSSLGSLEHLDLSYNYLSNLSSSWFKPL    123
TLR10       LRVLILCHN-RIQ-------------QLDLKTFEFNKELRYLDLSNNRLKSVT---WYLL    116
TLR1        LRILIISHN-RIQ-------------YLDISVFKFNQELEYLDLSHNKLVKIS---CHPT    113
TLR6        LTVLRLSHN-RIQ-------------LLDLSVFKFNQDLEYLDLSHNQLQKIS---CHPI    120
            *  : .                     :          *  * *  . :  :

                                               C183  C189
TLR3        TNLTELHLMSNSIQKIKNNPFVK--------------------------------QKNLI    150
TLR9        KSLISLSLSHTNILMLDSASLAGLHALRFLFMDGNCYYKNPCRQALEVAPGALLGLGNLT    202
TLR7        PSLQLLSLEANNIFSIRKENLTELANIEILYLGQNCYYRNPCYVSYSIEKDAFLNLTKLK    207
TLR8        ESLTELSLIQNNIYNITKEGISRLINLKNLYLAWNCYFNKVCEK-TNIEDGVFETLTNLE    222
TLR5        FHLFELRLYFCGL------------------------------SDAVLKDGYFRNLKALT    148
TLR4        SSLQKLVAVET--------------------------------NLASLENFPIGHLKTLK    153
TLR2        --------------------------------------------------------SSLT    127
TLR10       --------------------------------------------------------AGLR    120
TLR1        --------------------------------------------------------VNLK    117
TLR6        --------------------------------------------------------VSFR    124
                                                                      :

TLR3        TLDLSHNGLSSTKLGTQVQLENLQELLLSNNKIQALKSEELDIFANSSLKKLELSSNQIK    210
TLR9        HLSLKYNNLTVVP---RNLPSSLEYLLLSYNRIVKLAPEDLANLTA--LRVLDVGG----    253
TLR7        VLSLKDNNVTAVP---TVLPSTLTELYLYNNMIAKIQEDDFNNLNQ--LQILDLSG----    258
TLR8        LLSLSFNSLSHVP---PKLPSSLRKLFLSNTQIKYISEEDFKGLIN--LTLLDLSG----    273
TLR5        RLDLSKNQIRSLY-----------------------LHPSFGKLNS--LKSIDFSSNQIF    183
TLR4        ELNVAHNLIQSFK-----------------------LPEYFSNLTN--LEHLDLSSN---    185
TLR2        FLNLLGNPYKTLG-----------------------ETSLFSHLTK--LQILRVGNMDTF    162
TLR10       YLDLSFNDFDTMP-----------------------ICEEAGNMSH--LEILGLSGA---    152
TLR1        HLDLSFNAFDALP-----------------------ICKEFGNMSQ--LKFLGLSTT---    149
TLR6        HLDLSFNDFKALP-----------------------ICKEFGNLSQ--LNFLGLSAM---    156
             *.:  *                                    :    *  : ..
```

```
          C260 C263       C270 C273
TLR3      EFSPGCFHAIGRLFGLFLNN-VQLGPSLTEKLCLELANTSIRNLSLSNSQL-STTSNTTF   268
TLR9      -NCRRCDHAPNPCMECPRHFPQ-LHPDT------FSHLSRLEGLVLKDSSL-SWLNASWF   304
TLR7      -NCPRCYNAPFPCAPCKNNSPLQIPVNA------FDALTELKVLRLHSNSL-QHVPPRWF   310
TLR8      -NCPRCFNAPFPCVPCDGGASINIDRFA------FQNLTQLRYLNLSSTSL-RKINAAWF   325
TLR5      LVCE----HELEPLQGK----------------------TLSFFSLAANSLYSRVSVDWG   217
TLR4      -KIQSIYCTDLRVLHQM----------------------PLLNLSLDLSLNPMNF-IQ-P   220
TLR2      TKIQRKDFAGLTFLE-------------------------EL----EIDASDLQS-YE-P   191
TLR10     -KIQKSDFQKIAHLH-------------------------LNTVFLGFRTL--PH-YE-E   182
TLR1      -HLEKSSVLPIAHLN-------------------------ISKVLLVLGETYGEK-ED-P   181
TLR6      -KLQKLDLLPIAHLH-------------------------LSYILLDLRNYYIKE-NE-T   188

TLR3      LGLKWTNLT----MLDLSYNNLN--VVGNDSFAWLPQLEYFFLEYNNIQHLFSHSL----   318
TLR9      RGL--GNLR----VLDLSENFLYKCITKTKAFQGLTQLRKLNLSFNYQKRVSFAHLSLAP   358
TLR7      KNI--NKLQ----ELDLSQNFLAKEIGDAKFLHFLPSLIQLDLSFNFELQVYRASMNLSQ   364
TLR8      KNM--PHLK----VLDLEFNYLVGEIASGAFLTMLPRLEILDLSFNYIKGSYPQHINISR   379
TLR5      KCMNPFRNMVLEI-LDVSGNGWTVDIT----------------------GNFSNAISKSQ   254
TLR4      GAFKEIRLHKLTL----RNNFDSLNVMKT-CIQGLAGLEVHRL----VLGEFRNE-GNLE   270
TLR2      KSLKSIQNV-SHLILHMKQHILLLEIFVD-VTSSVECLELRDT----DLDT--------F   237
TLR10     GSLPILNTTKLHIVLPMDTNFW--VLLRD-GIKTSKILEMTNI----DGKS---------   226
TLR1      GGLQDFNTESLHIVFPTNKEFH--FILDV-SVKTVANLELSNI----KCVL--ED-SKCS   231
TLR6      ESLQILNAKTLHLVFHPTSLFA--IQVNI-SVNTLGCLQLTNI----K--L--ND-DNCQ   236
            :   .

TLR3      --HGLFNVRYLNLKRSFTKQSISLASLPKIDDFSFQWLKCLEHLN-MEDNDIPGIKSNMF   375
TLR9      SFGSLVALKELDMHGIF------FRSLDETTLRPLARLPMLQTLR-LQMNFINQAQLGIF   411
TLR7      AFSSLKSLKILRIRGYV------FKELKSFNLSPLHNLQNLEVLD-LGTNFIKIANLSMF   417
TLR8      NFSKLLSLRALHLRGYV------FQELREDDFQPLMQLPNLSTIN-LGINFIKQIDFKLF   432
TLR5      AFSLILA------HHIM-GAGFGFHNIKDPDQ----------------------------   279
TLR4      KFDKSA---LEGLCNLT-IEEFRLAYLDYYLDDIIDLFNCLTNVSSFSLVSVTIERVKDF   326
TLR2      HF-SELS---TGETNSL-IKKFTFRNVKITDESLFQVMKLLNQIS---------------   277
TLR10     QFVSYEMQRNLSLEHAK-TSVLLLNKVDLLWDDLFLILQFVWHTS---------------   270
TLR1      YFLSILAK---LQTNPK-LSSLTLNNIETTWNSFIRILQLVWHTT---------------   272
TLR6      VFIKFLSE---LTRGPT-LLNFTLNHIETTWKCLVRVFQFLWPKP---------------   277
                                  :  :

                                     C445
TLR3      TGLINLKYLSLSNSFTSLRT-L--------------------------------------   396
TLR9      RAFPGLRYVDLSDNRISGASELTATMGEADGGEKV-WLQPG-------DL----------   453
TLR7      KQFKRLKVIDLSVNKISPSGDSSEVGFCSNARTSVESYEPQVL-EQLHYFRYDKY-----   471
TLR8      QNFSNLEIIYLSENRISPLVKDTRQSYANSSSF-----QRHIRKRRSTDFEFDPH-----   482
TLR5      -------------NTFAGL--------------------------------------ARS   288
TLR4      SYNFGWQHLELVNCKFGQFPTLKL---------------------KS-------------   352
TLR2      ----GLLELEFDDCTLNGVGNFRA---------------------SDNDRVIDPGKVETL   312
TLR10     -----VEHFQIRNVTFGGKAYLDH---------------------NS--FDYSNTVMRTI   302
TLR1      -----VWYSSISNVKLQGQ--LDF---------------------RD--FDYSGTSLKAL   302
TLR6      -----VEYLNIYNLTIIES--IRE---------------------ED--FTYSKTTLKAL   307

                    C475            C491                          C521
TLR3      ------------------TNETFVSLAHSPLHILNLTKNKISKIESDAFSWLGHLEVLDL   438
TLR9      ----------APAPVDTPSSEDFRPNCSTLNFTLDLSRNNLVTVQPEMFAQLSHLQCLRL   503
TLR7      -------ARSCRFKNKEASFMSVNESCYKYGQTLDLSKNSIFFVKSSDFQHLSFLKCLNL   524
TLR8      -------SNFYHF-----TRPLIKPQCAAYGKALDLSLNSIFFIGPNQFENLPDIACLNL   530
TLR5      SVRHLDLSHGFVFSLN-----SRVFETLKDLKVLNLAYNKINKIADEAFYGLDNLQVLNL   343
TLR4      ------------------------------LKRLTFTSNK--GGNAFSEVDLPSLEFLDL   380
TLR2      TIRRLHIPRFYLFYDL-----STLYSLTERVKRITVENSKVFLVPCLLSQHLKSLEYLDL   367
TLR10     KLEHVHFRVFYI--QQ-----DKIYLLTKMDIENLTISNAQMPHMLFPNYPTKFQYLNF    355
TLR1      SIHQVVSDVEGF--PQ-----SYIYEIFSNMNIKNFTVSGTRMVHMLCPSKISPFLHLDF   355
TLR6      TIEHITNQVFLF--SQ-----TALYTVFSEMNIMMLTISDTPFIHMLCPHAPSTFKFLNF   360
                                             .  .              :  * :
```

```
TLR3            GLNEIGQELTG----QEWRGLENIFEIYLSYNKYLQLTRNSFALVPSLQRLMLRR----V    490
TLR9            SHNCISQAVNG----SQFLPLTGLQVLDLSHNKLDLYHEHSFTELPRLEALDLSYNSQPF    559
TLR7            SGNLISQTLNG----SEFQPLAELRYLDFSNNRLDLLHSTAFEELHKLEVLDISSNSHYF    580
TLR8            SANSNAQVLSG----TEFSAIPHVKYLDLTNNRLDFDNASALTELSDLEVLDLSYNSHYF    586
TLR5            SYNLLGELYSS-----NFYGLPKVAYIDLQKNHIAIIQDQTFKFLEKLQTLDLRDNALTT    398
TLR4            SRNGLS---FKGCCSQSDFGTTSLKYLDLSFNGVIT-MSSNFLGLEQLEHLDFQHSNLKQ    436
TLR2            SENLMVEEYLKNSACED--------------------------AWPSLQTLILRQNHLAS    401
TLR10           ANNILTDELFKRT---L--------------------------QLPHLKTLILNGNKLET    386
TLR1            SNNLLTDTVFENC---G--------------------------HLTELETLILQMNQLKE    386
TLR6            TQNVFTDSIFEKC---S--------------------------TLVKLETLILQKNGLKD    391
                  *                                            *: * :

TLR3            ALKNVDSSPSPFQPLRNLTILDLSNNNIANIN-DDMLEGLEKLEILDLQHNNLARLWKHA    549
TLR9            GMQGVGHNFSFVAHLRTLRHLSLAHNNIHSQ--VSQQLCSTSLRALDFSGNALGHMWAEG    617
TLR7            QSEGITHMLNFTKNLKVLQKLMMNDNDISSS--TSRTMESESLRTLEFRGNHLDVLWREG    638
TLR8            RIAGVTHHLEFIQNFTNLKVLNLSHNNIYTLT-DKYNLESKSLVELVFSGNRLDILWNDD    645
TLR5            I-HFIPSIP----------DIFLSGNKLVTLPKINLTANLIHLSE---N--------R-L    435
TLR4            M-SE-----------------------------FSVFLSLRNLIYLDISH-------T-H    458
TLR2            L-EK-----------------------------TGET-----------------------    408
TLR10           L-SL-----------------------------VSCF-----------------------    393
TLR1            L-SK-----------------------------IAEM-----------------------    393
TLR6            L-FK-----------------------------VGLM-----------------------    398

TLR3            NPGGPIYFLKGLSHLHILNLESNGFDEIPVEVFKDLF-----------------------    586
TLR9            D--LYLHFFQGLSGLIWLDLSQNRLHTLLPQTLRNLPKSLQVLRLRDNYLA-----FFKW    670
TLR7            DN-RYLQLFKNLLKLEELDISKNSLSFLPSGVFDGMPPNLKNLSLAKNGLK-----SFSW    692
TLR8            DN-RYISIFKGLKNLTRLDLSLNRLKHIPNEAFLNLPASLTELHINDNMLK-----FFNW    699
TLR5            ENLDILYFLLRVPHLQILILNQNRFSSCSGDQTPSENPSLEQLFLGENMLQLAWETELCW    495
TLR4            TRVAFNGIFNGLSSLEVLKMAGNSFQENFLPDIFTEL-----------------------    495
TLR2            --------LLTLKNLTNIDISKNSF--HSMPETCQWPEKMKYLNLSSTRI---------H    449
TLR10           --------ANN-TPLEHLDLSQNLLQ-HKNDENCSWPETVVNMNLSYNKL--------SD    435
TLR1            --------TTQMKSLQQLDISQNSVSYDEKKGDCSWTKSLLSLNMSSNIL--------TD    437
TLR6            --------TKDMPSLEILDVSWNSLESGRHKENCTWVESIVVLNLSSNML--------TD    442
                              *  : :  * .

                   C697                     C721
TLR3            ---------ELKIIDLG-----------------------LNNLNTLPASVFNNQVSLKS    614
TLR9            WSLHFL-PKLEVLDLAGNQLKALTNGSLPAGTRLRRLDVSCNSISFVAPGFFSKAKELRE    729
TLR7            KKLQCL-KNLETLDLSHNQLTTVPERLSNCSRSLKNLILKNNQIRSLTKYFLQDAFQLRY    751
TLR8            TLLQQF-PRLELLDLRGNKLLFLTDSLSDFTSSLRTLLLSHNRISHLPSGFLSEVSSLKH    758
TLR5            DVFEGL-SHLQVLYLNHNYLNSLPPGVFSHLTALRGLSLNSNRLTVLSH--NDLPANLEI    552
TLR4            --------RNLTFLDLSQCQLEQLSPTAFNSLSSLQVLNMSHNNFFSLDTFPYKCLNSLQV    548
TLR2            SVTGCIPKTLEILDVSNNNLNLF-S---LNLPQLKELYISRNKLMTLPD--ASLLPMLLV    503
TLR10           SVFRCLPKSIQILDLNNNQIQTV-PKETIHLMALRELNIAFNFLTDLPG--CSHFSRLSV    492
TLR1            TIFRCLPPRIKVLDLHSNKIKSI-PKQVVKLEALQELNVAFNSLTDLPG--CGSFSSLSV    494
TLR6            SVFRCLPPRIKVLDLHSNKIKSV-PKQVVKLEALQELNVAFNSLTDLPG--CGSFSSLSV    499
                         :  : :                          * :  :          *

                                                  C787 789
TLR3            LNLQKNLITSVEKKVFGP-AFRNLTELDMRFNPFDCTCESIAWFVNWINE-THTNIPELS    672
TLR9            LNLSANALKTVDHSWFGP-LASALQILDVSANPLHCACG-AAFMDFLLE--VQAAVPGLP    785
TLR7            LDLSSNKIQMIQKTSFPENVLNNLKMLLLHHNRFLCTCD-AVWFVWWVNH-TEVTIPYLA    809
TLR8            LDLSSNLLKTINKSALETKTTTKLSMLELHGNPFECTCD-IGDFRRWMDEHLNVKIPR-L    816
TLR5            LDISRNQLLAPNPDVFV-----SLSVLDITHNKFICECE-LSTFINWLNHT-NVTIAGPP    605
TLR4            LDYSLNHIMTSKKQELQH-FPSSLAFLNLTQNDFACTCE-HQSFLQWIKDQRQLL--VEV    604
TLR2            LKISRNAITTFSKEQLDS-FH-TLKTLEAGGNNFICSCE-FLSFTQE-QQALAKVLIDWP    559
TLR10           LNIEMNFILSPSLDFVQS-CQ-EVKTLNAGRNPFRCTCE-LKNFIQ-LETYSEVMMVGWS    548
TLR1            LIIDHNSVSHPSADFFQS-CQ-KMRSIKAGDNPFQCTCE-LGEFVKNIDQVSSEVLEGWP    551
TLR6            LIIDHNSVSHPSADFFQS-CQ-KMRSIKAGDNPFQCTCE-LREFVKNIDQVSSEVLEGWP    556
                *  . * :   .   .       :  :    * : * *     :    .
```

```
                C814                                 C833
TLR3     SHYLCNTPPHYHGFPVRLFDTS--SCKDSAPFELFFMINTSILLIFIFIVLLIHFEGWRI    730
TLR9     SRVKCGSPGQLQGLSIFAQDLR--LCLDEALSWDCFALSLLAVALGLGVPMLHHLCGWDL    843
TLR7     TDVTCVGPGAHKGQSVISLDLY--TCELDLTNLILFSLSISVSLFLMVMMTASHLYFWDV    867
TLR8     VDVICASPGDQRGKSIVSLELT--TCVSDVTAVILFFFTFFITTMVMLAALAHHLFYWDV    874
TLR5     ADIYCVYPDSFSGVSLFSLS--TEGCDEEEVLKSLKFSLFIVCT---VTLTLFLMTILTV    660
TLR4     ERMECATPSDKQGMPVLSLNI---TCQMNKTIIGVSVLSVLVVS---VVAVLVYKFYFHL    658
TLR2     ANYLCDSPSHVRGQQVQDVRLSVSECHRTALVSGMCCALFLL-----ILLTGVLCHRFHG    614
TLR10    DSYTCEYPLNLRGTRLKDVHLHELSCNTALLIVTIVVIMLVL-----GLAVAFCCLHFDL    603
TLR1     DSYKCDYPESYRGTLLKDFHMSELSCNITLLIVTIVATMLVL-----AVTVTSLCIYLDL    606
TLR6     DSYKCDYPESYRGSPLKDFHMSELSCNITLLIVTIGATMLVL-----AVTVTSLCIYLDL    611
             *   *    *   :         *

                 C874                            C889 890
TLR3     SFYWNVSVHRVLGFKEID-R------QTEQFEYAAYIIHAYKD---KDWVWEHFSSMEKE    780
TLR9     WYCFHLCLAWLPWRGRQSGR------DEDALPYDAFVVFDKTQSAVADWVYNELRGQLEE    897
TLR7     WYIYHFCKAKIKGYQRLI---------SPDCCYDAFIVYDTKDPAVTEWVLAELVAKLED    918
TLR8     WFIYNVCLAKVKGYRSLS---------TSQTFYDAYISYDTKDASVTDWVINELRYHLEE    925
TLR5     TKFRGFCFICYKTAQRLVFKDHPQGTEPDMYKYDAYLCFSSKD---FTWVQNALLKHLDT    717
TLR4     ----MLLAGCIKY--G-----------RGENIYDAFVIYSSQD---EDWVRNELVKNLEE    698
TLR2     LWYMKMMWAWLQA--KRKPRKAP----SRNICYDAFVSYSERD---AYWVENLMVQELEN    665
TLR10    PWYLRMLGQCTQT--WHRVRKTTQEQLKRNVRFHAFISYSEHD---SLWVKNELIPNLEK    658
TLR1     PWYLRMVCQWTQT--RRRARNIPLEELQRNLQFHAFISYSGHD---SFWMKNELLPNLEK    661
TLR6     PWYLRMVCQWTQT--RRRARNIPLEELQRNLQFHAFISYSEHD---SAWVKSELVPYLEK    666
               .                             : *:: .   :     *:   :    .

                    C927
TLR3     ----DQSLKFCLEERDFEAGVFELEAIVN-SIKRSRKIIFVITHHLLKDPLCKRFKVHHA    835
TLR9     CR-GRWALRLCLEERDWLPGKTLFENLWA-SVYGSRKTLFVLAHTDRVSGLLR-ASFLLA    954
TLR7     PR-EK-HFNLCLEERDWLPGQPVLENLSQ-SIQLSKKTVFVMTDKYAKTENFK-IAFYLS    974
TLR8     SR-DK-NVLICLEERDWDPGLAIIDNLMQ-SINQSKKTVFVLTKKYAKSWNFK-TAFYLA    981
TLR5     QYSDQNRFNICFEERDFVPGENRIANIQD-AIWNSRKIVCLVSRHFLRDGWCL-EAFSYA    775
TLR4     G---VPPFQLCLHYRDFIPGVAIAANIIHEGFHKSRKVIVVVSQHFIQSRWCI-FEYEIA    754
TLR2     F---NPPFKICLHKRDFIPGKWIIDNIID-SIEKSHKTVFVLSENFVKSEWCK-YELDFS    720
TLR10    E---DGSILICLYESYFDPGKSISENIVS-FIEKSYKSIFVLSPNFVQNEWCH-YEFYFA    713
TLR1     E---G--MQICLHERNFVPGKSIVENIIT-CIEKSYKSIFVLSPNFVQSEWCH-YELYFA    714
TLR6     E---D--IQICLHERNFVPGKSIVENIIN-CIEKSYKSIFVLSPNFVQSEWCH-YELYFA    719
                . :*:    :  *        :    .  * * : :::                :

                                                  C1008              C1028
TLR3     VQQAIEQNLDSIILVFLEEIPDYKLNHALCLRRGMFKSHCILNWPVQKERIGAFRHKLQV    895
TLR9     QQRLLEDRKDVVVLVILSPD--GRRSRYVRLRQRLC-RQSVLLWPHQPSGQRSFWAQLGM    1011
TLR7     HQRLMDEKVDVIILIFLEKP--FQKSKFLQLRKRLC-GSSVLEWPTNPQAHPYFWQCLKN    1031
TLR8     LQRLMDENMDVIIFILLEPV--LQHSQYLRLRQRIC-KSSILQWPDNPKAEGLFWQTLRN    1038
TLR5     QGRCLSDLNSALIMVVVGSLSQYQLMKH-QSIRGFVQKQQYLRWPEDLQDVGWFLHKLSQ    834
TLR4     QTWQFLSSRAGIIFIVLQKVEKTLLRQQ-VELYRLLSRNTYLEWEDSVLGRHIFWRRLRK    813
TLR2     HFRLFDENNDAAILILLEPIEKKAIPQRFCKLRKIMNTKTYLEWPMDEAQREGFWVNLRA    780
TLR10    HHNLFHENSDHIILILLEPIPFYCIPTRYHKLKALLEKKAYLEWPKDRRKCGLFWANLRA    773
TLR1     HHNLFHEGSNSLILILLEPIPQYSIPSSYHKLKSLMARRTYLEWPKEKSKRGLFWANLRA    774
TLR6     HHNLFHEGSNNLILILLEPIPQNSIPNKYHKLKALMTQRTYLQWPKEKSKRGLFWANIRA    779
             : .     :::.:                :       * *  .        *  :

TLR3     ALGSKNSVH------------------------------  904
TLR9     ALTRDNHHFYNRNFCQGPTAE------------------  1032
TLR7     ALATDNHVAYSQVFKETV---------------------  1049
TLR8     VVLTENDSRYNNMYVDSIKQY------------------  1059
TLR5     QILKKEKEKKKDNNIPLQT-------VATIS-------  858
TLR4     ALLDGKSWNPEGTV-GTGCNWQE---ATSI--------  839
TLR2     AIKS-----------------------------------  784
TLR10    AINVNVLATREMYELQTFTELNEESRGSTISLMRTDCL  811
TLR1     AINIKLTEQAKK---------------------------  786
TLR6     AFNMKLTLVTENNDVKS----------------------  796
           .
```

Supplementary Figure 9 Multiple sequence alignment analysis demonstrating the position of all cysteine residues on human TLR7. Individual sequences of human TLRs were obtained from NCBI GenBank protein databases with the following accession numbers TLR1 (CAG38593.1), TLR2 (AAH33756.1), TLR3 (ABC86910.1), TLR4 (AAF07823.1), TLR5 (AAI09119.1), TLR6 (BAA78631.1), TLR7 (AAZ99026.1), TLR8 (AAZ95441.1), TLR9 (AAZ95520.1) and TLR10 (AAY78491.1) and then sequence alignment was performed with CLUSTAL OMEGA (EMBL-EBI). Shown in red dotted rectangular boxes are the cysteines on human TLR7 and the respective position indicated.

```
                                                                                C36
[Salmo      MLSRMT------RSECASFHVCGVILLGLWCSSVLAAGSWYPKTLPCDVTLDSNDTMVNV  54
[Xenopus    MHGKTFKV-----FYFGMRRQLLFFLISILSFSGLLATNWFPKSLPCDVEQNAKGNVIVV  55
[Gallus     -MTNLSEVAAHRKMVHHARTSNALLFVLLFLFPMLLSGRWFPKTLPCDVEA--FESTVRV  57
[Mus        ---------MV--FSMWTRKRQILIFLNMLLVSRVFGFRWFPKTLPCEVKVNIPEAHVIV  49
[Rattus     ---------MV--FPMWTLKRQSFIFLNMILVSRVLGFRWYPKTLPCDVSLDSTNTHVIV  49
[Homo       ---------MV--FPMWTLKRLILILFNIILISKLLGARWFPKTLPCDVTLDVPKNHVIV  49
[Sus        ---------MV--FPVWTLKRQFLILFNIVLISELLGARWFPKTLPCDVSLDAPNAHVIV  49
[Bos        -MGDLFLYFQV--FPMWTLKRQFPILFNMILISGLLGARWFPKTLPCDVTLDAPNTHVIV  57
                                   ::. :      :  .   *:**:***:*         : *
             C51                                                     C98 C100
[Salmo      DCTERGLLEVPKDIPRNTTNLTLTINHIPHINSTSFQGLENLTEIDMRCNCVPIKIGPKD  114
[Xenopus    DCSDRHLTSIPWGIPTNVTNLTLTINHIPRISVDSFAEFTNLVELDFRCNCVPAKVGPKD  115
[Gallus     DCSDRRLKEVPRGIPGNATNLTLTINHIPRISPASFTQLENLVEIDFRCNCVPPRLGPKD  117
[Mus        DCTDKHLTEIPEGIPTNTTNLTLTINHIPSISPDSFRRLNHLEEIDLRCNCVPVLLGSKA  109
[Rattus     DCTDKHLTEIPEGIPTNTTNLTLTINHIPSISPDSFHRLKHLEELDLRCNCVPILLGSKA  109
[Homo       DCTDKHLTEIPGGIPTNTTNLTLTINHIPDISPASFHRLDHLVEIDFRCNCVPIPLGSKN  109
[Sus        DCTDKHLTAIPGGIPTNATNLTLTINHIASITPASFQQLDHLVEIDFRCNCIPVRLGPKD  109
[Bos        DCTDKHLTEIPGGIPANATNLTLTINHIAGISPASFHRLDHLVEIDFRCNCVPVRLGPKD  117
            **:::  *   :*   ** *.**********  *.   **   :  .*  *:*:****:*   :*  *
              C112
[Salmo      RMCTESVTIKTNTFKDLRNLKALYLDGNQLSSIPKGLPPNLILLSLEVNKIYTILKRNLS  174
[Xenopus    HVCTKRLDVEDRSFASLYNLRSLYLDGNQLIEFPKGLPPNLQLLSLEINNIISISRNNLS  175
[Gallus     NVCVTPPSIENGSFAALTRLKSLYLDANQLSKIPRGLPATLRLLSLEANNIFSIKKNTFS  177
[Mus        NVCTKRLQIRPGSFSGLSDLKALYLDGNQLLEIPQDLPSSLHLLSLEANNIFSITKENLT  169
[Rattus     NVCTKRLQIRPGSFSGLSDLKSLYLDGNQLLEIPQDLPSSLQLLSLEANNIFSITKENLS  169
[Homo       NMCIKRLQIKPRSFSGLTYLKSLYLDGNQLLEIPQGLPPSLQLLSLEANNIFSIRKENLT  169
[Sus        NICTRRLQIKPSSFSKLTYLKALYLDGNQLLEIPRDLPPSLQLLSLEANNIFWIMKENLT  169
[Bos        NVCTKRLQIKPNSFSKLTYLKSLYLDGNQLLEIPQDLPPSLQLLSLEANNIFLIMKENLT  177
            .:*      :.   :*   *   *::****.***  .:*:  **  .*  ***** *:*   *  :..::
                          C183  C189
[Salmo      DITNVQILYLGQNCYYRNPCNVSYQIEEGAFLQLGNMTLLSLKSNNLSYIPPRLPTSLRE  234
[Xenopus    ELSNIQMLYLGQNCYHRNPCSDSFKIEKDAFKDLKNLSILSMKSNNLSFVPGGLSDSLKE  235
[Gallus     ELRNIELLYLGQNCYYRNPCNVSFEIEETAFLNLKNLTVLSLKSNNLTFIPPNLSSTLKE  237
[Mus        ELVNIETLYLGQNCYYRNPCNVSYSIEKDAFLVMRNLKVLSLKDNNVTAVPTTLPPNLLE  229
[Rattus     ELVNIESLYLGQNCYYRNPCNVSYSIEKDAFLVMKNLKVLSLKDNNVTAVPTILPPNLLE  229
[Homo       ELANIEILYLGQNCYYRNPCYVSYSIEKDAFLNLTKLKVLSLKDNNVTAVPTVLPSTLTE  229
[Sus        ELANLEMLYLGQNCYYRNPCNVSFSIEKDAFLSLRNLKLLSLKDNNISAVPTVLPSTLTE  229
[Bos        ELANLEILYLGQNCYYRNPCNVSFTIEKDAFLNMRNLKLLSLKDNNISAVPTVLPSSLTE  237
            :: *::  *********:****  *:  **:  **   :  :::.:**:*.**::  :*   *   .* *
                                             C260 C263  C270 C273
[Salmo      LYLYNNKIEMVTDKDFHNLTQLEILDLCGNCPRCYNAPFRCIRCPNN-SLQIDPSTFKTL  293
[Xenopus    LYLYNNAIQYIEEHDLENLINLEILDLSGNCPRCYNSPFRCTRCPNNAPIQIHPKAFSSL  295
[Gallus     LYIYNNRIQEVQEHDLSNLYNLEILDLSGNCPRCYNAPYRCTRCPNI-SIKIHSKAFYSL  296
[Mus        LYLYNNIIKKIQENDFNNLNELQVLDLSGNCPRCYNVPYRCTRCENNSPLQIHDNAFNSL  289
[Rattus     LYLYNNIIKRIQEHDFNKLSQLQVLDLSGNCPRCYNVPYRCTRCENNSPLQIHDNAFDSL  289
[Homo       LYLYNNMIAKIQEDDFNNLNQLQILDLSGNCPRCYNAPFRCARCKNNSPLQIPVNAFDAL  289
[Sus        LFLYNNIIAKIQEDDFNNLSQLQVLDLSGNCPRCYNVPFRCTRCENNAPLQIHLHAFDAL  289
[Bos        LYLYNNIITKIQEDDFNNLSQLQVLDLSGNCPRCYNVPFRCTRCENNSPLQIDPNAFDAL  297
            *::*** *   :  :.*:  :*  :*::***.************ *:*** ** *    ::*   :*  :*

[Salmo      TKLRILRLHSNSLTYVLREWFQNCKELRVLDLSTNFLAREIAITYFPRALPNLEELDLSF  353
[Xenopus    KNLQVLRLHSNSLRSIPEQWFKNNRNLQVLDLSENFLASEISTANFLKYIPSLKSLDLSF  355
[Gallus     KKLRILRLHSNSLQSIPSSWFKNIKNLKNLDLSQNFLIKEIGDAEFLKLIPSLVELDLSF  356
[Mus        TELKVLRLHSNSLQHVPPTWFKNMRNLQELDLSQNYLAREIEEAKFLHFLPNLVELDFSF  349
[Rattus     TELKVLRLHSNSLQHVPAEWFKNMSNLQELDLSQNYLAREIEEAKFLNSLPNLVQLDLSF  349
[Homo       TELKVLRLHSNSLQHVPPRWFKNINKLQELDLSQNFLAKEIGDAKFLHFLPSLIQLDLSF  349
[Sus        TELQVLRLHSNSLQYVPQRWFQNLNKLKELDLSQNFLAKEIGDAKFLHLLPNLVKLDLSF  349
[Bos        TELQVLRLHSNSLQHVPQRWFKNINKLKELDLSQNFLAKEIGDAKFLHLLHNLVNLDLSF  357
            .:*::********   :    **:*   :*:  **** *:*   **   :  *  .  :  .*  .**:**

[Salmo      NYELQRYPATLHLSPSFSSLKSLKVLRIRAFVFQQLTLEDISPLIHLKNLEVIDLGTNFI  413
[Xenopus    NFELQVYPSDLKLSSIFSSLASLETLRIRGYVFQNLKKNNLMPLVHLPNLTLLDLSTNFI  415
[Gallus     NFELQMYSPFLNLSKTFSCLSNLETLRIKGYVFKELREENLDPLLNLRNLTVLDLGTNFI  416
[Mus        NYELQVYHASITLPHSLSSLENLKILRVKGYVFKELKNSSLSVLHKLPRLEVLDLGTNFI  409
[Rattus     NYELQVYHASITLPHSLSSLTKLKNLYIKGYVFKELKDSSLSVLHNLSNLEVLDLGTNFI  409
[Homo       NFELQVYRASMNLSQAFSSLKSLKILRIRGYVFKELKSFNLSPLHNLQNLEVLDLGTNFI  409
[Sus        NYELQVYHTFMNLSDSFSSLKNLKVLRIKGYVFKELKSLNLSPLRNLPNLEVLDLGTNFI  409
[Bos        NYDLQVYHAVINLSDAFSSLKKLKVLRIKGYVFKELNSLNLFPLHNLPNLEVLDLGTNFI  417
            *::** *    :  *    :*.*  .*:  *  ::..:**::*     .:   *  .*  .*  ::**.****
```

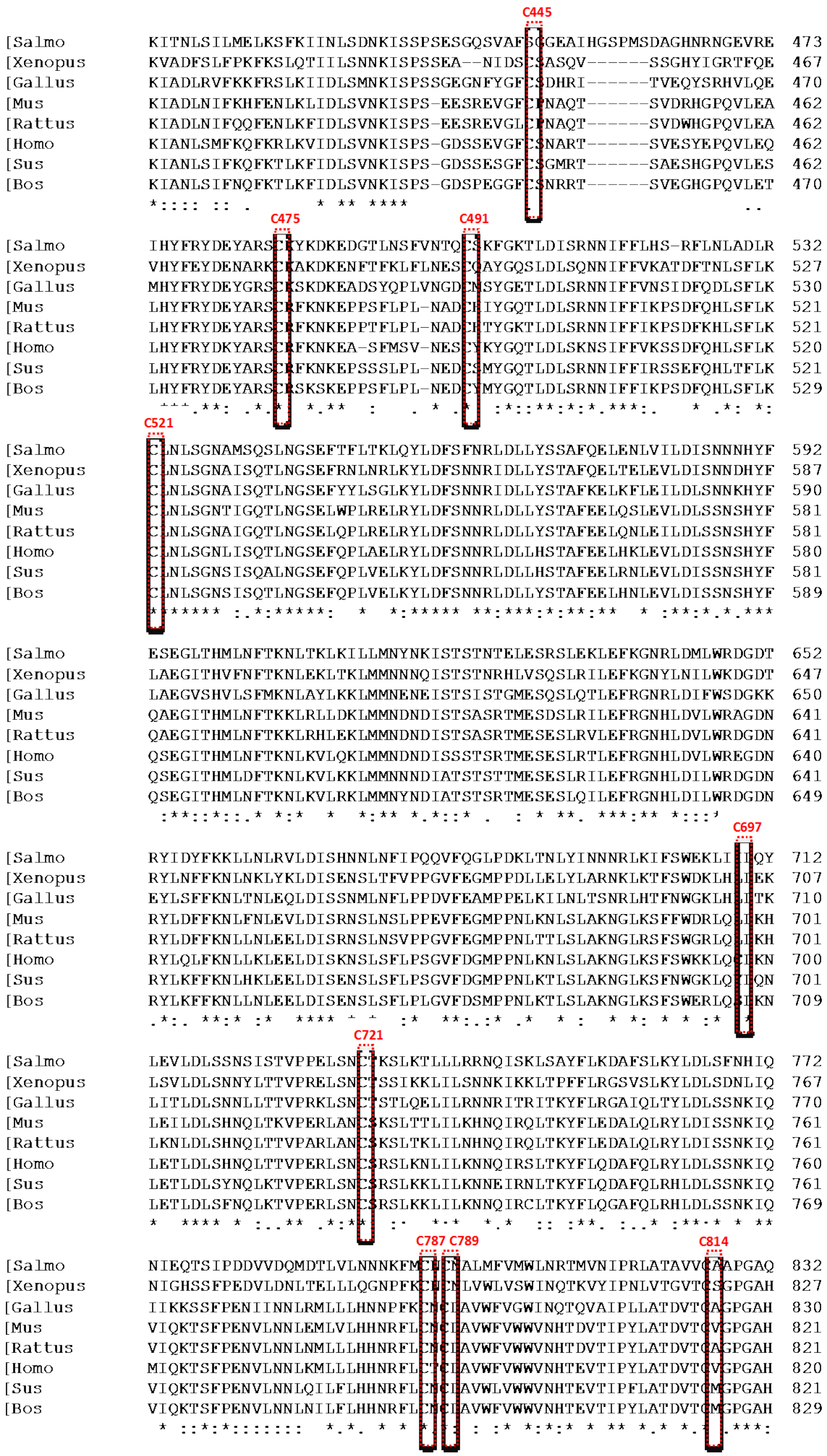
C445
C475
C491
C521
C697
C721
C787 C789
C814

```
[Salmo        RGHPVISLDLELQACQHNYLSIILYILLTSLVLSFVTLPISSHLFLWDVWYLYHFLLAKF 892
[Xenopus      RGQSLVLLDL--YTCEQYHLNLILQALSASFIICLMVVSVSSHLFYWDFWFIYHLFKAKI 885
[Gallus       KGRSLVFLDL--NTCELDTSYFIMYALSTSAVLCLMMFAVMSHLYFWDVWYSYHYCTAKL 888
[Mus          KGQSVISLDL--YTCELDLTNLILFSVSISSVLFLMVVMTTSHLFFWDMWYIYYFWKAKI 879
[Rattus       KGQSVISLDL--YTCELDLTNLILFSVSISSVLFLMIVMTTSHLFFWDMWYIYYFWKAKI 879
[Homo         KGQSVISLDL--YTCELDLTNLILFSLSISVSLFLMVMMTASHLYFWDVWYIYHFCKAKI 878
[Sus          KGQSVVSLDL--YTCELDLTNFVLFSLSLSAVLFLIVITIANHLYFWDVWYSYHFCKAKI 879
[Bos          KGQSVVSLDL--YTCELDLTNFILFSLSISAVLSLMMITIANHLYFWDVWYSYHFCKAKI 887
              :*: :: .:.  .    ::: :  *  : :: .  .**:    *:   **:

                        C8898 C890                                 C927
[Salmo        KGYRRLSSPSAAYDAFVVYDKKDPEVSEWVLKELLVQLEEHGDHPLQICLEERDWIPGCP 952
[Xenopus      HGYKRF--PKCCYDALIMYDTKDSAVSDWVFNDLVNILEKQGNKMLNICLEERDFLAGQP 943
[Gallus       KGYRRIPLPIACYDAFIAYDNTDLAVNEWVMTELVEKLEDQKARQFNICLEERDWLPGQP 948
[Mus          KGYQHLQSMKSCYDAFIVYDTKNSAVTEWVLQELVAKLEDPREKHFNICLEERDWLPGQP 939
[Rattus       KGYQHLQSMKSCYDAFIVYDTKNSAVTEWVLQELVVKLEDPREKHFNICLEERDWLPGQP 939
[Homo         KGYQRLISPDCCYDAFIVYDTKDPAVTEWVLAELVAKLEDPREKHFNICLEERDWLPGQP 938
[Sus          KGYQRLISPNSCYDAFIVYDTKDPAVTEWVLDELVAKLEDPREKHFNICLEERDWLPGQP 939
[Bos          KGYRRLISPNSCYDAFIVYDTKDPAVTEWVLDELVAKLEDPREKCFNICLEERDWLPGQP 947
              :**:::    .:. :**:: **..: *.:**: :*:  **.   : ::******:: * *

[Salmo        LIDNLSQSIHQSKRTVFILTNKYIKSGDFKTAFYMAHQRLMDERDDVIVLIFLEKVPSHS 1012
[Xenopus      FLDNLSESIQISRKTVFVLTRKYVKKGHFKTAFYMAHQRLIEEKVDVIILILLEKTLQRS 1003
[Gallus       VFDNLSQSIQLSKKTIFVLTNKYIKSGTFKTTFYMAHQRLLDEKIDVIILIFLEKVLQKS 1008
[Mus          VLENLSQSIQLSKKTVFVMTQKYAKTESFKMAFYLSHQRLLDEKVDVIILIFLEKPLQKS 999
[Rattus       VLENLSQSIQLSRKTVFVMTQKYAKTESFKMAFYLSHQRLMDEKVDVIILIFLEKPLQKS 999
[Homo         VLENLSQSIQLSKKTVFVMTDKYAKTENFKIAFYLSHQRLMDEKVDVIILIFLEKPFQKS 998
[Sus          VLENLSQSIQLSKKTVFVMTDKYAKTEKFKIAFYLSHQRLMDEKVDVIILIFLEKPLQKS 999
[Bos          VLENLSQSIQLSKKTVFVMTDKYAKTENFKIAFYLSHQRLMDEKVDVIILIFLEKPLQKS 1007
              .::***:      :*:*::* ** *.      *::****::*: ***:**:***   .:*

                       C1008                  C1029
[Salmo        KYLRLRKRIYRRSVIEWPTNPQAQQYFWKSLRSVLVTDSQKQYSNLFKETR      1063
[Xenopus      RYLRLRKRICANSVLYWPSNPNSQSYFWHCLKSAIATENQMAYDKLFKDHT      1054
[Gallus       RYVQLRKRICRSSVLEWPTNPRSQPYFWQRLKNAIAMNNTLSYNKLLQETV      1059
[Mus          KFLQLRKRICRSSVLEWPANPQAHPYFWQCLKNALTTDNHVAYSQMFKETV      1050
[Rattus       KFLQLRKRICSSSVLEWPTNPQAHPYFWQCLKNALTTDNHVAYSQMFKETV      1050
[Homo         KFLQLRKRICGSSVLEWPTNPQAHPYFWQCLKNALATDNHVAYSQVFKETV      1049
[Sus          KFFQLRKRICGSSVLEWPTNPQAHPYFWQCLKNALATDNHVTYSQVFKETA      1050
[Bos          KFLQLRKRICGSSVLEWPTNPQAHPYFWQCLKNALATDNHVTYSQVFKETA      1058
              :::.:*****  **: **:**.:: ***  *:..:. :.  *.:::::
```

Supplementary Figure 10. Multiple sequence alignment analysis of vertebrate TLR7. Individual sequences of TLRs were obtained from NCBI GenBank protein databases with the following accession numbers *Salmo salar* (**CCX35457.1**), *Xenopus tropicalis* (**AAI66280.1**), *Gallus gallus* (**ACR26243.1**), *Mus musculus* (**AAI32386.1**), *Rattus norvegius* (**NP_001091051.1**), *Homo sapiens* (AAZ99026.1), *Sus scrofa* (**ABQ52583.1**) and *Bos taurus* (**NP_001028933.1**) and then sequence alignment was performed with CLUSTAL OMEGA (EMBL-EBI). Shown in red dotted rectangular boxes are the cysteines on human TLR7 and the respective position indicated.

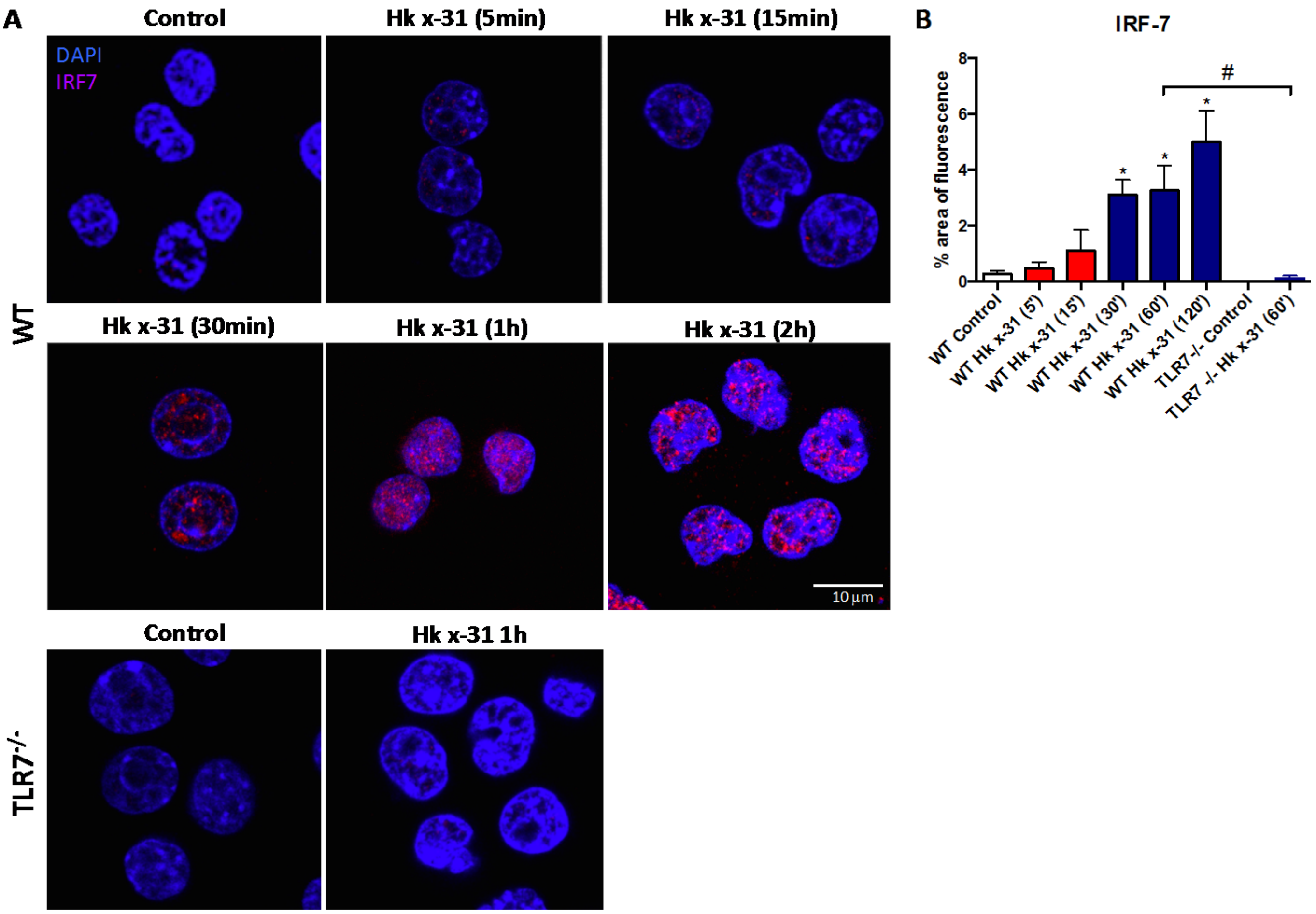

Supplementary Figure 11. Influenza A virus causes interferon regulatory factor 7 (IRF-7) nuclear translocation *via* a TLR7-dependent mechanism.

(A) Immortalized bone marrow macrophages from WT or $TLR7^{-/-}$ mice were either left untreated or infected with HKx31 influenza A virus (HKx31, MOI of 10) for various times as indicated and then labeled with the IRF-7 antibody and 4',6'- diamidino-2-phenylindole (DAPI). Images are representative of >100 cells analyzed over 4 separate experiments. Original magnification X100. All data are represented as mean ± SEM. One-way ANOVA followed by Dunnett's *post hoc* test for multiple comparisons. *P<0.05 compared to WT controls. #P<0.05 - comparison indicated by horizontal bar.

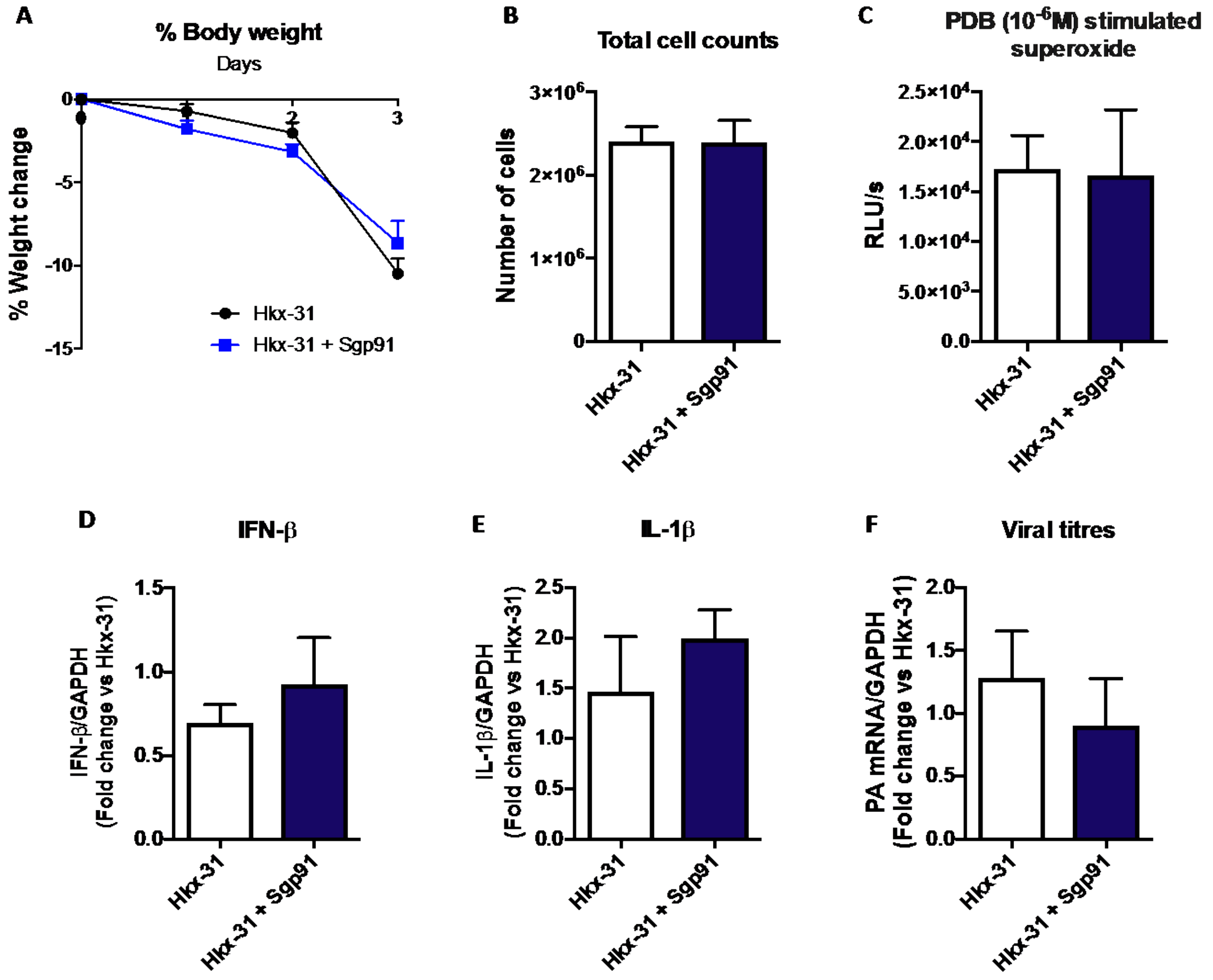

Supplementary Figure 12. Scrambled cholestanol-conjugated gp91ds-TAT failed to influence influenza A virus dependent pathology in vivo.

Scrambled gp91ds-TAT (Sgp91; 0.02mg/kg/day) was delivered intranasally to WT mice once daily for 4 days. At 24h after the first dose of inhibitor, mice were infected with HKx31 influenza A virus (1×10^5 PFU per mouse). Mice were culled at day 3 post-infection and **(A)** body weight, **(B)** airway inflammation was assessed by BALF cell counts, **(C)** superoxide was measured from BALF inflammatory cells via L-O12 (100µM)-enhanced chemiluminescence, (D and E) lung IFN-β and IL-1β mRNA, respectively, was determined by QPCR and **(F)** viral load was determined byQPCR. All data are represented as mean ± SEM, n=8.

TLR7-H	C36	C51	C98	C100	C112	C183	C189	C260	C263	C270	C273	C445	C475	C491	C521	C697	C721	C787	C789	C814	C833	C874	C889	C890	C927	C1008	C1028	Accession
TLR1-H	-	-	-	-	-	-	-	-	-	-	-	-	-	-	-	-	Yes	Yes	Yes	Yes	-	Yes	-	-	Yes	-	-	CAG38593.1
TLR2-H	-	-	-	-	-	-	-	-	-	-	-	-	-	-	-	Yes	-	Yes	Yes	Yes	-	-	-	Yes	Yes	-	-	AAH33756.1
TLR3-H	-	-	-	-	-	-	-	-	Yes	-	-	-	-	-	-	-	-	Yes	Yes	Yes	yes	-	-	-	Yes	-	-	ABC86910.1
TLR4-H	-	-	-	-	-	-	-	-	Yes	-	-	-	Yes	-	Yes	-	-	Yes	Yes	Yes	Yes	-	-	-	Yes	-	-	AAF07823.1
TLR5-H	-	-	-	-	-	-	-	-	-	-	-	-	-	-	-	-	-	Yes	Yes	Yes	Yes	Yes	-	-	Yes	-	-	AAI09119.1
TLR6-H	-	-	-	-	-	-	-	-	-	-	-	-	-	-	-	-	Yes	Yes	Yes	Yes	-	Yes	-	-	Yes	-	-	BAA78631.1
TLR8-H	Yes	Yes	-	-	-	Yes	Yes	Yes	Yes	Yes	Yes	-	-	Yes	Yes	-	-	Yes	Yes	Yes	Yes	Yes	-	-	Yes	Yes	-	AAZ99441.1
TLR9-H	Yes	Yes	-	Yes	Yes	Yes	Yes	Yes	Yes	Yes	Yes	-	-	Yes	Yes	-	-	Yes	Yes	Yes	Yes	Yes	-	-	Yes	Yes	-	AAZ95520.1
TLR10-H	-	-	-	-	-	-	-	-	-	-	-	-	-	-	-	Yes	-	Yes	Yes	Yes	-	-	-	-	Yes	-	-	AAY78491.1
TLR7 *Mus*	Yes	Yes	Yes	Yes	Yes	Yes	Yes	Yes	Yes	Yes	Yes	Yes	Yes	Yes	Yes	-	Yes	Yes	Yes	Yes	Yes	-	-	Yes	Yes	Yes	Yes	AAI32386.1
TLR7 *rattus*	Yes	Yes	Yes	Yes	Yes	Yes	Yes	Yes	Yes	Yes	Yes	Yes	Yes	Yes	Yes	-	Yes	Yes	Yes	Yes	Yes	-	-	Yes	Yes	Yes	Yes	NP_001091051.1
TLR7 *Sus*	Yes	Yes	Yes	Yes	Yes	Yes	Yes	Yes	Yes	Yes	Yes	Yes	Yes	Yes	Yes	-	Yes	Yes	Yes	Yes	Yes	Yes	-	Yes	Yes	Yes	Yes	ABQ52583.1
TLR7 *Bos taurus*	Yes	Yes	Yes	Yes	Yes	Yes	Yes	Yes	Yes	Yes	Yes	Yes	Yes	Yes	Yes	-	Yes	Yes	Yes	Yes	Yes	Yes	-	Yes	Yes	Yes	Yes	NP_001028933.1
TLR7 *Xenopus*	Yes	Yes	Yes	Yes	Yes	Yes	Yes	Yes	Yes	Yes	Yes	Yes	Yes	Yes	Yes	-	Yes	Yes	Yes	Yes	Yes	-	Yes	Yes	Yes	Yes	Yes	AAI66280.1
TLR7 *Gallus*	Yes	Yes	Yes	Yes	Yes	Yes	Yes	Yes	Yes	Yes	Yes	Yes	Yes	Yes	Yes	-	Yes	Yes	Yes	Yes	Yes	Yes	-	Yes	Yes	Yes	-	ACR26243.1
TLR7 *Salmo*	Yes	Yes	Yes	Yes	Yes	Yes	Yes	Yes	Yes	Yes	Yes	-	Yes	Yes	Yes	-	Yes	Yes	Yes	Yes	Yes	-	-	-	Yes	-	-	CCX35487.1

Supplementary Table 1. Pairwise sequence alignment analysis of human TLR7 vs other members of the human TLR family and vs vertebrate TLR7. Individual sequences of TLRs were obtained from NCBI GenBank protein databases with the indicated accession numbers and then sequence alignment was performed with Pubmed NCBI BLAST.

Gene	Company	Gene expression assay	Catalog no.	Ref Seq
**Mouse IL-1β TaqMan Primer**	Applied Biosystems	Mm00434228_m1	4331182	NM_008361.3
**Mouse CYBB TaqMan Primer**	Applied Biosystems	Mm01287743_m1	4331182	NM_007807.5
**Mouse IFNB1 TaqMan Primer**	Applied Biosystems	Mm00439552_s1	4331182	NM_010510.1
**Mouse TNFα TaqMan Primer**	Applied Biosystems	Mm00443258_m1	4331182	NM_001278601.1
**Mouse IL6 TaqMan Primer**	Applied Biosystems	Mm00446190_m1	4331182	NM_031168.1
**Mouse TLR7 TaqMan Primer**	Applied Biosystems	Mm00446590_m1	4331182	NM_016562.3
**Mouse GAPDH (X20)**	Applied Biosystems		4352339E	
**Influenza polymerase forward primer**	Applied Biosystems	5′-CGGTCCAAATTCCTGCTGA-3′		
**Influenza polymerase reverse primer**	Applied Biosystems	5′-CATTGGGTTCCTTCCATCCA-3-3′		

Supplementary Table 2: List of primers and their sources and reference sequences. The influenza polymerase primers were custom synthesized and the sequences are shown.
